# Systemic inflammatory response syndrome and organ dysfunctions are early predictors for ICU readmission

**DOI:** 10.1186/cc10207

**Published:** 2011-06-22

**Authors:** AM Japiassú, H Falcão, AP Coscia, DM Costa, RBN Silva

**Affiliations:** 1 Hospital Quinta D'Or, Rio De Janeiro - RJ, Brazil

## Introduction

Previous studies have indicated risk factors for ICU re-admission; sepsis, respiratory insufficiency, medical admission, organ dysfunctions and age are associated with this outcome. Specific physiological and laboratory data were explored in some studies, but no association was shown with readmission. Our hypothesis is that inflammation and organ dysfunctions are more important for this outcome than demographic data or type of admission.

## Methods

We selected all consecutive patients admitted to the five ICUs of a tertiary hospital. All patients discharged from the ICU at least once were included. Demographic, physiological and laboratory data were collected on the first day after the first admission and organ support resources (mechanical ventilation, use of vasopressors and renal dialysis) were researched throughout the ICU stay. Organ dysfunctions were defined as they are in the SOFA score. A logistic regression was made with all of the parameters with *P *< 0.2 in the univariate analysis.

## Results

There were 1,073 patients admitted to all five ICUs during the study period. Seventy patients died during the first admission and were excluded, resulting in the analysis of 1,003 patients. There were 160 ICU readmissions from 130 patients. The readmission rate was 13%. Sepsis and respiratory or cardiovascular decompensation were the most common causes of readmission. ICU readmitted patients were more likely to be older (median 75 × 69 years, *P *= 0.004), medical rather than surgical type (70 × 61%, *P *= 0.04), originated from the ward or intermediate care unit (15 × 6%, *P *< 0.001), with any infection on ICU admission (38 × 29%, *P *= 0.03), and higher Charlson index (1 × 0 point, *P *< 0.001).

## Conclusion

Systemic inflammatory response syndrome with organ dysfunctions are predictors for ICU readmissions, despite the patient's origin, type of admission and the presence of infection at admission.

